# Predictive Value of Lung Ultrasound Combined With ACEF Score for the Prognosis of Acute Myocardial Infarction

**DOI:** 10.1002/clc.70111

**Published:** 2025-03-03

**Authors:** Okasha Tahir, Ali Bin Nasir, Sonam Lohana, Taha Naveed, Muhammad Abdullah

**Affiliations:** ^1^ Bacha Khan Medical College Mardan Pakistan; ^2^ Karachi Institute of Medical Sciences Karachi Pakistan; ^3^ Liaquat University of Medical and Health Sciences Jamshoro Pakistan; ^4^ Basic Health Unit Sehna Gujrat Punjab Pakistan; ^5^ HITEC Institute of Medical Sciences Taxilla Pakistan

**Keywords:** ACEF score, acute myocardial infarction, lung ultrasound

## Conflicts of Interest

The authors declare no conflicts of interest.

Dear Editor,

We read the recent study by Lun et al. [1] on the “Predictive value of lung ultrasound (LUS) combined with the ACEF score for the prognosis of acute myocardial infarction (AMI)” with great interest. The authors provide a novel approach to risk stratification, yet several methodological and clinical concerns warrant discussion.

The study's exclusion of patients older than 80, those with significant pulmonary disease, and those without LUS within 48 h of admission substantially limits its external validity. Elder patients and those with chronic lung conditions constitute a substantial proportion of AMI cases, and their exclusion raises concerns about the findings' real‐world applicability [2, 3]. The single‐center design with a relatively small sample size (*n* = 204) further restricts generalizability. By addressing this limitation, future research could improve the external validity of LUS as a prognostic tool for a broader patient population.

While the study suggests that combining LUS with the ACEF score improves predictive performance, it does not adequately account for key confounders. For instance, diuretic use was significantly associated with adverse outcomes (OR 4.79, *p* < 0.01), yet its impact on B‐line counts and overall prognostication was not thoroughly explored. Without rigorous adjustment, the study may overestimate the independent predictive value of LUS [3]. A more rigorous multivariate analysis or propensity score matching would strengthen the study's conclusions and ensure that LUS retains its predictive value independent of other clinical interventions.

The median follow‐up period of 12 months is insufficient to capture long‐term cardiovascular outcomes, particularly for AMI patients at risk of late heart failure events. Additionally, the reliance on telephone follow‐ups introduces potential reporting bias, as clinical outcomes were not objectively verified through imaging or biomarker assessments [4]. Extending the follow‐up period and incorporating objective clinical data would enhance the reliability of LUS and ACEF score‐based prognostication.

Despite these limitations, the study introduces an important concept by integrating LUS into AMI risk stratification. Future studies with more extensive, multicenter cohorts, improved statistical adjustments, and extended follow‐up are necessary to confirm the robustness of this approach. We commend the authors for their valuable contribution to the evolving landscape.

## Data Availability

The authors have nothing to report.
